# Acute phase proteins as local biomarkers of respiratory infection in calves

**DOI:** 10.1186/s12917-015-0485-7

**Published:** 2015-07-25

**Authors:** Annette Prohl, Wieland Schroedl, Heidrun Rhode, Petra Reinhold

**Affiliations:** Institute of Molecular Pathogenesis at ‘Friedrich-Loeffler-Institut’ (Federal Research Institute for Animal Health), Naumburger Str. 96a, 07743 Jena, Germany; Institute of Bacteriology and Mycology, Veterinary Faculty at The University of Leipzig, Leipzig; An den Tierkliniken 29, 04103 Leipzig, Germany; Institute of Biochemistry I, University Hospital Jena, Nonnenplan 2, 07743 Jena, Germany

**Keywords:** *Chlamydia psittaci*, Acute phase proteins, Lung, Blood, Broncho-alveolar lavage, Bovine, Respiratory infection

## Abstract

**Background:**

Cumulating reports suggest that acute phase proteins (APPs) do not only play a role as systemic inflammatory mediators, but are also expressed in different tissues as local reaction to inflammatory stimuli. The present study aimed to evaluate presence and changes in luminal lung concentrations of the APPs haptoglobin (Hp), lipopolysaccharide binding protein (LBP), C-reactive protein (CRP), and lactoferrin (Lf) in calves with an acute respiratory disease experimentally induced by *Chlamydia (C.) psittaci*.

**Results:**

Intra-bronchial inoculation of the pathogen resulted in a consistent respiratory illness. In venous blood of the infected calves (*n* = 13), concentrations of plasma proteins and serum LBP were assessed (i) before exposure and (ii) 8 times within 14 days after inoculation (dpi). Increasing clinical illness correlated significantly with increasing LBP—and decreasing albumin concentrations in blood, both verifying a systemic acute phase response.

Broncho-alveolar lavage fluid (BALF) was obtained from all 13 calves experimentally infected with *C. psittaci* at 4, 9 and 14 dpi, and from 6 uninfected healthy calves. Concentrations of bovine serum albumin (BSA), Hp, LBP, CRP and Lf in BALF were determined by ELISA. In infected animals, absolute concentrations of LBP and Hp in BALF correlated significantly with the respiratory score. The quotient [LBP]/[BSA] in BALF peaked significantly in acutely infected animals (4 dpi), showed a time-dependent decrease during the recovery phase (9-14 dpi), and was significantly higher compared to healthy controls. Concentrations of Hp and Lf in BALF as well as [Hp]/[BSA]—and [Lf]/[BSA]-quotients decreased during the study in infected animals, but were never higher than in healthy controls. CRP concentrations and [CRP]/[BSA]-quotient did not express significant differences between infected and healthy animals or during the course of infection.

**Conclusion:**

In conclusion, absolute concentrations of LBP in blood and BALF as well as the quotient [LBP]/[BSA] in BALF perfectly paralleled the clinical course of respiratory illness after infection. Beside LBP, the suitability of Hp and Lf as local biomarkers of respiratory infections in cattle and their role in the local response to pathogens is worth further investigation, while CRP does not seem to play a role in local defense mechanisms of the bovine lung.

## Background

Acute phase proteins (APPs) have been studied as markers of disease in animals for decades (reviewed for example in [1, 2]). Most research groups studied the concentrations of APPs in blood, but there are also attempts to use them as local markers of inflammation.

Already in 1986, a first hint on the extra-hepatic expression of the serum amyloid family was reported [3] followed by the description of the expression of several APPs on the mRNA-level in a variety of tissues of mice. In 2009, a rapid and widely disseminated APP expression in lymphatic organs has been shown in pigs on the mRNA-level after experimental bacterial infection, occurring concomitantly with the hepatic response [4]. Following these, the list of singularly described APPs that have been identified to be extra-hepatically expressed in a regulated manner is constantly growing.

So, exemplarily, serum amyloid A (SAA) is now known to be constitutively expressed in various normal extra-hepatic tissues and cancers in humans [5], horses, and cattle [6]. After bacterial challenge or milk stasis, focal expression of SAA3 was dramatically up-regulated in the udder of dairy cows [7, 8]. Maximal milk concentrations of this protein were similar to the ones in blood plasma [9].

Haptoglobin (Hp) is expressed in human salivary gland, muscle [10], lung [11], and skin [12, 13]. Arterial expression of Hp is enhanced after balloon dilatation and pertains to a unique set of glycoforms different from the synthesis product of the liver [14]. In cattle the mRNA of this protein has been found constitutively in mammary gland, leukocytes [15], and 33 further tissues with comparatively high concentrations in the exocrine pancreas and the submandibular salivary gland [16]. The expression of Hp could be shown to increase more than 80-fold in mammary gland cells after focal udder infection of dairy cows [8]. This could explain that the concentration of Hp in milk increases several hours earlier than in blood [17]. Moreover, Hp is also released into bronchial fluid in respiratory infection of pigs [18] and cattle [19], and into saliva. Salivary Hp is proposed to serve as early biomarker for porcine virus infections [20] and as a stress marker [21].

Lipopolysaccharide binding protein (LBP) is known to be expressed in humans in oral and periodontal tissues [22], in gastrointestinal epithelia, salivary and mammary gland [23] and in respiratory type II epithelial cells [22]. In cows it was shown in several organs and tissues, among them also the lung, salivary and mammary glands [23] and adipose tissue [24]. Moreover, like Hp, this protein is considerably induced in mammary gland cells in response to bacterial infection in heifers [9].

Many studies are available on serum concentrations of APPs after respiratory infection (for cattle, this is reviewed in [25]), but data of their concentrations in the lung as the site of infection and inflammation are scarce. In young pigs, influenza infection causes increased APP concentrations in broncho-alveolar lavage fluid (BALF), which are probably secreted by the respiratory tract epithelia. Here, concentrations increased steeply for LBP and moderately for C reactive protein (CRP) and Hp, the latter two showing higher maximal concentrations in blood serum [18]. A study in calves found higher concentrations of Hp and SAA in BALF supernatant of calves with bronchopneumonia than in healthy calves [26]. Taken together, this led to the hypothesis that due to their production in the lung, some APPs are suitable local biomarkers for respiratory inflammation. To address this, it is necessary to determine APP concentrations not only in blood, but also in BALF. From former studies in a bovine model of respiratory infection with *Chlamydia (C.) psittaci* and *Parachlamydia (P.) acanthamoebae* we knew that serum levels of some APPs increase after inoculation with these pathogens [27–34]. Prior to this study, data of LBP, Hp, lactoferrin (Lf), and CRP levels in blood of animals enrolled in former studies with the above mentioned model were summarized and are presented in Table 1. Briefly, after inoculation with *C. psittaci,* LBP and Hp increased significantly, and after inoculation with *P. acanthamoebae* we observed a significant increase of LBP and Lf in blood sera.

**Table 1:** Concentrations of LBP, Lf, Hp, and CRP in the blood sera of calves

APP	Healthy animals	Number	Infection with *Chlamydia psittaci* (2–3 dpi)	Number	Infection with *Parachlamydia acanthamoebae* (1 dpi)	Number	Studies calves were enrolled in
LBP [μg × mL^−1^]	6.0 (1.0–69.8)	123	136.6*** (25.9–470.0)	80^a^	15.0*** (3.1–75.6)	21^c^	[27, 31, 34]
Lf [ng × mL^−1^]	146.3 (71.4–345.8)	43	NA		190.1** (72.0–393.5)	21^c^	[27]
Hp [μg × mL^−1^]	5.8 (0.03–127.6)	69	29.9*** (0.9–3048.8)	35^b^	NA		[30, 28, 29, 33]
CRP [μg × mL^−1^]	47.8 (8.3–178.7)	69	56.1 (13.6–147.7)	35^b^	NA		[30, 28, 29, 33]

*LBP* lipopolysaccharide binding protein; *Lf* lactoferrin; *Hp* haptoglobin; *CRP* C-reactive protein. Values are given as median (minimum-maximum). Age, origin and breed of the calves and methods of marker detection were identical with this study. Data related to infected animals are given at that time point (day post inoculation (dpi)), where they were maximal within an observation period of 2 weeks after inoculation. For statistical evaluation, values from the day with the highest values were compared to pre-inoculation values of the same animals (Mann–Whitney-*U* test). **0.001 ≤ *P* < 0.01; *** *P* < 0.0001. ^a^animals inoculated with 10^8^ inclusion forming units (ifu); ^b^animals inoculated with 10^6^, 10^7^, 10^8^, or 10^9^ ifu; ^c^animals inoculated with 10^10^ ifu. *NA* not analyzed

Aim of the present study was to evaluate the suitability of Lf, CRP, LBP, and Hp as local biomarkers in respiratory infections in cattle using a well-defined animal model. For this purpose, concentrations of APPs were determined in the BALF of *C. psittaci* inoculated calves at three time points after inoculation. Additionally, concentrations of LBP and globulin fractions in blood serum were determined to verify the systemic acute phase response in the animals. Correlations were assessed between measured APPs in both BALF and blood, and the corresponding scores representing the severity of clinical illness.

## Methods

### Legal conformity and ethics statement

This study was carried out in strict accordance with the German Animal Welfare Act. The protocol was approved by the Committee on the Ethics of Animal Experiments and the Protection of Animals of the State of Thuringia, Germany (Permit Numbers: 04–002/07 and 04–004/11). All experiments were done in a containment of biosafety level 2 under supervision of the authorized institutional Agent for Animal Protection. Bronchoscopy was strictly performed under general anesthesia in infected animals and under light sedation in uninfected controls. During the entire study, every effort was made to minimize suffering.

### Animals

In this prospective and controlled study, 19 conventionally raised calves (Holstein-Friesian, male) were included. Animals originated from one farm without any history of *Chlamydia*-associated health problems. Before the study, the herd of origin was regularly checked for the presence of *Chlamydiae* by the National Reference Laboratory for Psittacosis. Calves were purchased at the age of 15 to 27 days weighing between 47.2 and 71.2 kg. After a quarantine period of at least 21 days and confirmation of a clinically healthy status, animals were included in the study. Exclusion of co-infections and standard antibiotic treatment after purchase, *i.e.* about 3 weeks before challenge, was performed as described previously [31, 34]. Briefly, the presence of *Mycoplasma*, *Pasteurella*, or *Mannheimia* spp. was evaluated in nasal swabs taken immediately before challenge and before necropsy, as well as in lung tissue samples obtained during necropsy. Serology was used to check for antibodies against *Mycoplasma bovis*, bovine respiratory syncytial virus, parainfluenza 3 virus, adenovirus type 3, bovine herpes virus 1, and bovine virus diarrhea/mucosal disease virus. Results did not suggest the presence of respiratory co-infections in any animal included in this study.

Throughout the entire study, animals were reared under standardized conditions (room climate: 18-20 °C, rel. humidity: 60-65 %) and in accordance with international guidelines for animal welfare. Uninfected controls were housed separately from infected animals. Nutrition included commercial milk replacers and coarse meal. Water and hay were supplied *ad libitum*. None of the given feed contained antibiotics.

### Study design

#### Uninfected controls

Six calves served as uninfected controls. Beginning at the age of 3 months, BALF was sampled three times from each animal every two weeks. For bronchoscopy, animals were sedated with xylazine (Rompun 2 %, Bayer Vital GmbH, Leverkusen, Germany) and broncho-alveolar lavage was performed endoscopically in the standing animal. Five fractions of 20 mL sterile, isotonic, body warm saline were used as a flushing liquid, and recovered fluid was stored on ice in siliconized glass bottles until further preparation. The fluid recovery rate in this group was 60 % (median, range: 23 %). All animals were clinically healthy during the study.

#### Infected animals

The 13 infected animals in the present study had served as untreated controls in previous studies [31, 34].

Inoculation with 10^8^ ifu *C. psittaci* was performed intrabronchially as described previously [32, 33]. At time point of inoculation, animals were aged 6–8 weeks. Animals were clinically examined on a daily basis throughout the whole study, starting one week before inoculation. Results were summarized using a previously published clinical scoring system [33]. The respiratory score was calculated by adding the points for respiratory rate, ocular and nasal discharge, coughing, and dyspnea.

**Fig. 1:**
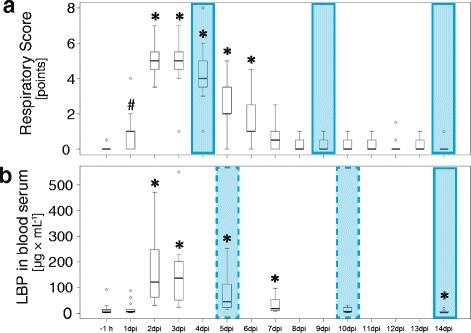
Kinetics of respiratory score and lipopolysaccharide-binding protein (LBP) in blood serum of calves. (**a**) Respiratory score and (**b**) concentration of LBP in blood serum of calves before and after inoculation with *Chlamydia (C.) psittaci*. Blue bars represent time-points of broncho-alveolar lavage and corresponding blood sampling. Blue panel with solid line: exact time point, blue panel with dashed line: blood collection one day after broncho-alveolar lavage. Post inoculation values were compared to the value 1 h before inoculation (−1 h) with the Wilcoxon signed rank test with Holm adjustment. -7 d: 7 days before inoculation; −4 d: 4 days before inoculation; dpi: days past inoculation; grey: animals prior to inoculation with *C. psittaci;* white: animals after inoculation with *C. psittaci* (*n* = 13). The 13 calves served as untreated controls in two previous treatment studies where the general clinical scores and concentrations of LBP in blood sera have been published separately [31, 34]

From all infected animals, blood was sampled 7 days, 4 days, and one hour before inoculation (ai) as well as 1, 2, 3, 5, 7, 10 and 14 days post inoculation (dpi) by puncture of the jugular vein into plastic syringes (S-Monovette, KABE LABORTECHNIK GmbH, Nümbrecht-Elsenroth, Germany). From the same animals, broncho-alveolar lavage fluid (BALF) was endoscopically sampled at 4 and 9 dpi under general anesthesia in 5 fractions of 20 mL as described elsewhere [32]. The sampling point 4 dpi represents the end of the acute phase of the disease, whereas the samples from 9 and 14 dpi represent the recovery phase (Fig. 1). All animals were euthanized 14 dpi and BALF was sampled from the exenterated lung in 6 fractions of 20 mL as described [31]. Sterile, isotonic, body warm saline was always used as flushing liquid. The fluid recovery rate in this group was 81 % (20 %) *in vivo* and 63 % (29 %) *ex vivo.*

### BALF preparation and analysis

BALF was centrifuged at 300 × *g* for 20 minutes; supernatant was decanted and stored at -80 °C until further analysis. Concentrations of LBP, CRP, Lf, and Hp in the supernatant were determined as described previously (LBP: [30], CRP and Lf: [35], Hp: [36]). For ELISA analyses, the dilution factors of BALF were 1:2 for CRP and LBP, 1:4 for Hp, and 1:200 or 1:4 for Lf.

The quantitative detection of bovine serum albumin (BSA) in BALF was done using the following ELISA. The working volume per well was 100 μl and the incubation time was one hour at room temperature on a microtiter plate shaker (400 rpm). ELISA-plates (96 well, flat-bottomed, high binding, Corning, New York, USA) were coated with 2 μg/ml IgG-anti-BSA (from sheep, affinity purified, Bethyl Laboratories Inc., Montgomery, Texas, USA) in 0.1 M NaHCO_3_. After incubation the plates were washed two times with 0.154 M NaCl and 0.05 % (v/v) Tween 20 (wash solution) by using a 12 canal microtiter plate washer (NUNC, Wiesbaden, Germany). The standard was bovine serum albumin (purity about 98 %, Sigma-Aldrich-Chemie Steinheim, Germany) in the concentration range between 80 and 1.25 ng/ml. The BALF samples were diluted 1:400 or higher. The assay buffer for dilution was phosphate-buffered saline (PBS according to Dulbecco, pH 7.35) with 0.2 % (v/v) Tween 20 and 10 mM Na_2_EDTA. After incubation and four times of washing with wash solution, the horseradish peroxidase conjugate of IgG-anti-BSA (from sheep, affinity purified, Bethyl Laboratories Inc., Montgomery, Texas, USA) was diluted in assay buffer to 1:20 000, and was added to the wells. The plates were incubated, washed four times, and the horseradish peroxidase was determined with the colorimetric substrate 3 mM H_2_O_2_ and 1 mM 3,3′,5,5′-tetramethylbenzidine in 0.2 M citrate buffer (pH 3.95). The reaction was stopped with 1 M H_2_SO_4_ (50 μl per well). The optical density (OD-value) was measured with a microplate-ELISA-reader at 450 nm. The standard curve (BSA standard-concentrations vs. OD-values) was determined using TableCurve software (Systat Software, Erkrath, Germany) and the concentrations of BSA in samples were then calculated by consideration of the dilution factor.

### Blood serum preparation and analysis

Serum was harvested from venous blood samples by centrifugation (1750 × g; 20 min), and was stored at −80 °C until analysis. Concentrations of total protein, BSA, α1-globulin, α2-globulin, β-globulin, and γ-globulin were determined as described elsewhere [28]. Concentrations of LBP in blood serum have been analyzed as previously described [33]. The animals included in the present study had served as untreated control animals in two previous treatment studies, where their corresponding blood-LBP concentrations have been published [31, 34]. For the study presented here, the two groups of infected but untreated calves have been combined to one infected group in order to exemplify the systemic APP response in comparison to the local one.

### Statistical methods

Statgraphics Centurion XVI (Version 16.1.18, Statistical Graphics Corporation) and R [37] were used for statistical analyses. Due to the small sample sizes, data were assumed not to be normally distributed and non-parametrical tests were chosen for statistical evaluation of all data.

BSA levels in BALF supernatant were used for normalization of probable expressed local marker concentrations, and the [local marker]/[BSA]-quotient was calculated.

The Wilcoxon signed rank test with zero handling according to Pratt from the package coin [38] with Holm adjustment was used for comparing pre—with post-inoculational values from blood samples. The sample obtained 1 h before inoculation was always used for comparison with post-inoculation values. For comparison of infected animals with uninfected controls, the two-sided Mann–Whitney *U* test with Holm adjustment was used. Values from BALF samples of infected animals were always compared to values from the first sample of the healthy controls, because the age difference was lowest at that time point. For comparing different time points of BALF samples in infected and in uninfected animals, Wilcoxon signed rank test with Holm adjustment was used. Values of *P* ≤ 0.05 were considered significant. Values of 0.05 ≤ *P* < 0.1 were regarded as tendencies and are given in the graphs. Unless stated differently, data are given as median and range. In ‘Box and Whiskers plots’, outlier values (circles) are more than 1.5 times of the length of a box away from the median.

Spearman Rank Correlations and multiple regression analyses were used to identify significant correlations between the severity of clinical signs and APPs assessed in BALF or blood, respectively.

## Results

### Clinical signs

Compared to baseline values, the respiratory score augmented significantly after inoculation in all animals, was maximal on 2 and 3 dpi, and remained significantly increased until 6 dpi (Fig. 1a). The respiratory score contributed to about 50 % to the general clinical score that followed the same time course (data not shown, compare [31]).

### LBP, albumin, and proteins assessed in blood sera

**Fig. 2:**
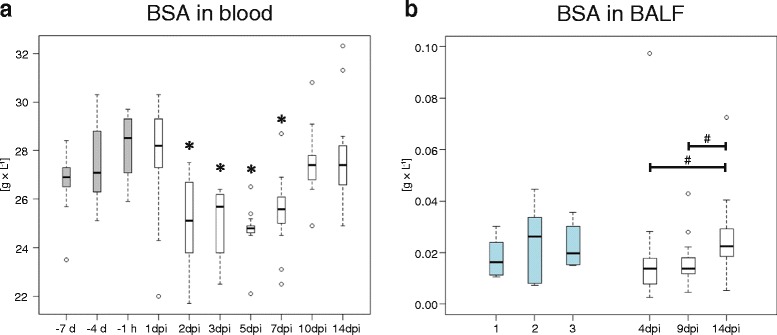
Bovine serum albumin (BSA) in calves. (**a**) BSA in blood sera of calves before and after inoculation with *Chlamydia (C.) psittaci*. Post inoculation blood values were compared to the value 1 h before inoculation (−1 h) with the Wilcoxon signed rank test with Holm adjustment. -7 d: 7 days before inoculation; −4 d: 4 days before inoculation; dpi: days past inoculation; grey: animals prior to inoculation with *C. psittaci;* white: animals after inoculation with *C. psittaci* (*n* = 13). (**b**) BSA in BALF supernatant of healthy and *C. psittaci-*infected calves. BALF values of infected animals were compared to the first sample from healthy controls, since at that time point the age difference between the animals was lowest (Mann–Whitney-*U* test with Holm adjustment). 1, 2, 3: first, second, and third sample from healthy controls; light blue: healthy control animals (*n* = 6); white: animals after inoculation with *C. psittaci* (*n* = 13). #: 0.05 ≤ *P* < 0.1; *: 0.01 ≤ *P* < 0.05

**Fig. 3:**
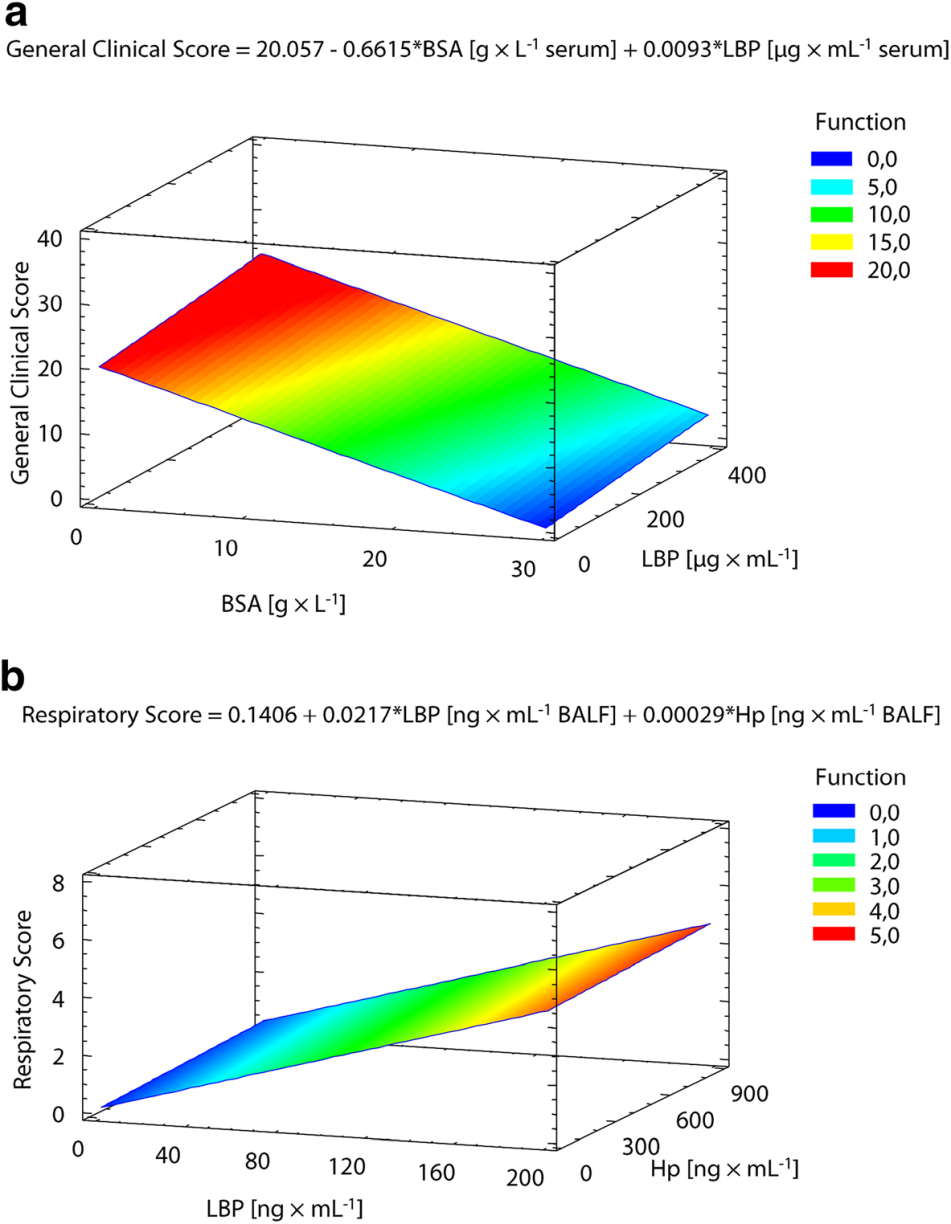
Relationships between clinical signs and markers of acute phase reaction assessed in blood (**a**) or BALF (**b**) in calves with an acute respiratory infection induced by *Chlamydia psittaci*. Models of multiple regressions (*n* = 39). **a** Dependent variable: General Clinical Score (z-axis). Independent variables: concentrations of bovine serum albumin (BSA; x-axis) and LBP (y-axis) in blood serum. R^2^ = 35.4 %. *P*-Value of the model = 0.0004. **b** Dependent variable: Respiratory Score (z-axis). Independent variables: concentrations of Lipopolysaccharide Binding Protein (LBP; x-axis) and Haptoglobin (Hp; y-axis) in broncho-alveolar lavage fluid (BALF). R^2^ = 55.3 %. *P*-Value of the model = 0.0000

The concentration of LBP in blood serum paralleled the clinical sings, being maximal in all animals on 2 and 3 dpi. It remained significantly increased until 7 dpi (Fig. 1b). In contrast, the concentration of BSA in the blood serum was significantly decreased from 2 to 7 dpi as compared to 1 h before inoculation (Fig. 2a). Rank correlations revealed that blood concentrations of both BSA and LBP were significantly linked to the general clinical score and the respiratory score (Table 2). The complex correlation between decreasing blood concentrations of BSA and increasing ones of LBP with a higher severity of the disease, based on multiple regressions, is illustrated in Fig. 3a.

**Table 2:** Significant Spearman Rank Correlations between the concentrations of BSA and LBP in blood serum and the respiratory score and general clinical score in calves with an experimentally induced respiratory *Chlamydia psittaci* infection (*n* = 39)

	BSA (blood)	LBP (blood)	General clinical score	Respiratory score
BSA (blood)		r_sp_ = −0.55	r_sp_ = −0.64	r_sp_ = −0.60
		*P* < 0.001	*P* < 0.001	*P* < 0.001
LBP (blood)	r_sp_ = −0.55		r_sp_ = 0.47	r_sp_ = 0.47
	*P* < 0.001		*P* = 0.004	*P* = 0.004
General Clinical Score	r_sp_ = −0.64	r_sp_ = 0.47		r_sp_ = 0.77
	*P* < 0.001	*P* = 0.004		*P* < 0.001
Respiratory Score	r_sp_ = −0.60	r_sp_ = 0.47	r_sp_ = 0.77	
	*P* < 0.001	*P* = 0.004	*P* < 0.001	

*BSA* bovine serum albumin. *LBP* lipopolysaccharide binding protein. r_sp_: Coefficient of Spearman Rank Correlation. *P*: probability level. *n* = 39*: all time points after exposure were included in analysis, i.e. the acute phase of respiratory illness (4 dpi; *n* = 13), and the recovery phase (9 dpi; *n* = 13 and 14 dpi; *n* = 13)

**Fig. 4:**
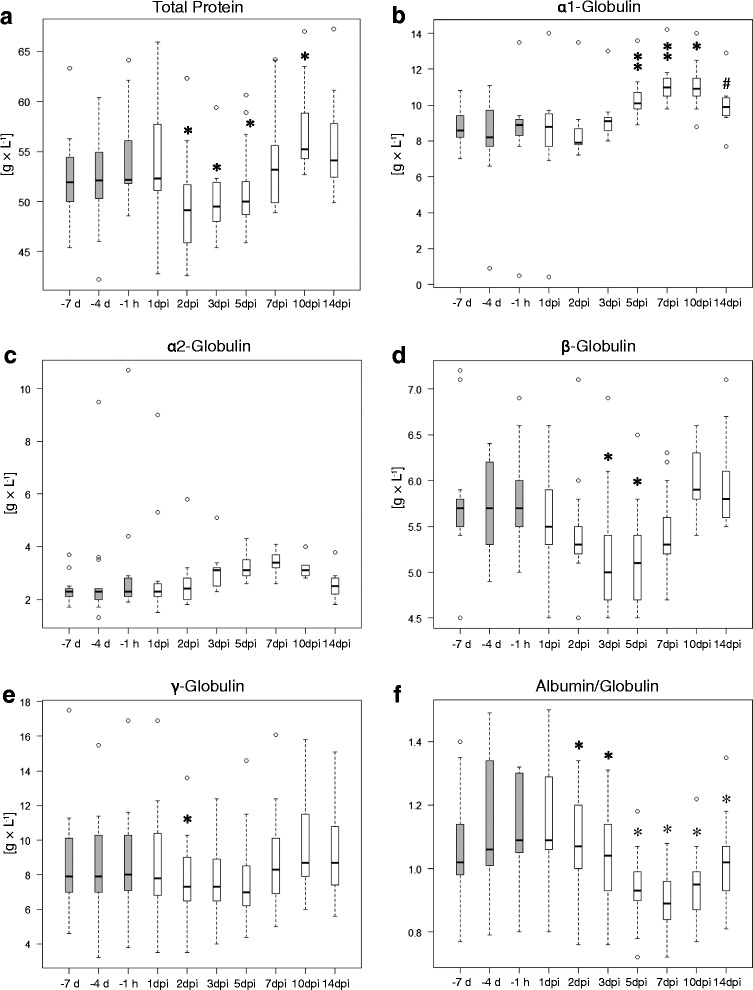
Protein fractions in blood sera of calves before and after inoculation with *Chlamydia psittaci*. Post inoculation values were compared to the value 1 h before inoculation (−1 h) with the Wilcoxon signed rank test with Holm adjustment. -7 d: 7 days before inoculation; −4 d: 4 days before inoculation; dpi: days post inoculation; grey: animals prior to inoculation with *Chlamydia (C.) psittaci;* white: animals after inoculation with *C. psittaci* (*n* = 13); #: 0.05 ≤ *P* < 0.1; *: 0.01 ≤ *P* < 0.05; ** 0.001 ≤ *P* < 0.01

Other blood proteins showed variable results summarized in Fig. 4. The total protein concentration in blood sera of calves dropped significantly 2 days after intrabronchial inoculation with *C. psittaci* (Fig. 4a). Baseline level was reached on 7 dpi, and protein concentration was significantly higher 10 dpi than before inoculation. The concentration of α1-globulins in blood serum remained stable until 3 dpi and was significantly increased from 5 to 10 dpi (Fig. 4b). On 14 dpi, levels of α1-globulins were not yet returned to baseline level. Concentrations of α2-globulins followed the same kinetics as concentrations of α1-globulins, but the changes were not statistically significant (Fig. 4c). Concentration of β-globulins in the blood serum started to drop after inoculation and was significantly lower than pre-inoculational values on 3 and 5 dpi (Fig. 4d). Baseline level was reached again 10 dpi. The amount of γ-globulins in the blood serum was slightly reduced after inoculation; changes were only significant 2 dpi (Fig. 4e). The albumin/globulin-quotient was significantly reduced from 2 to 14 dpi with a minimum on 7 dpi (Fig. 4f).

### LBP, Hp, Lf, CRP, and BSA assessed in BALF supernatant

Absolute concentrations of all potential APPs measured in BALF supernatant are given in Table 3. Interestingly, during the recovery phase (9 and 14 dpi), BALF-concentrations of Hp and Lf in animals inoculated with *C. psittaci* were significantly lower compared to both, healthy control calves and inoculated animals 4 dpi. In contrast, the absolute concentration of CRP in BALF supernatant was slightly, but not significantly higher in infected animals than in healthy controls.

**Table 3:** Concentrations of LBP, Hp, Lf, and CRP in the BALF supernatant of healthy calves and of calves with an experimentally induced respiratory *Chlamydia psittaci* infection

	LBP [ng × mL^−1^]	Hp [ng × mL^−1^]	Lf [μg × mL^−1^]	CRP [ng × mL^−1^]
Healthy controls	9.5 (1.7–23.6)	107.3 (3.0–347.0)	0.96 (0.4–2.8)	0.104 (0.0–0.3)
Infected 4 dpi	61.5 (12.4–172.9)*^,a^	12.3 (1.6–12554.8)^a^	0.38 (0.1–3.6)^a^	0.399 (0.0–7.9)
Infected 9 dpi	15.5 (5.5–35.3)^b^	11.6 (1.3–2120.9)^a^	0.26 (0.1–0.8)*	0.212 (0.0–0.8)
Infected 14 dpi	12.0 (2.5–24.1)^b^	0.7 (0.0–3.9)*^, b^	0.17 (0.1–1.0)*^,b^	0.523 (0.0–3.0)

Values are given as median (minimum-maximum). Concentrations for healthy controls (*n* = 6) are only given for the first sampling point. Asterisks indicate significant differences compared to healthy controls (Mann–Whitney-*U* test with Holm adjustment, *P < 0.05*). Different small letters (a,b) indicate significant differences between different time points in infected animals (Wilcoxon signed rank test with Holm adjustment, *P < 0.05*). Infected: animals inoculated with *C. psittaci* (*n* = 13); *dpi* days after inoculation; *BSA* bovine serum albumin; *LBP* lipopolysaccharide binding protein; *Hp* haptoglobin; *Lf* lactoferrin; *CRP* C reactive protein

With respect to the course of infection, LBP concentrations in BALF supernatant were significantly augmented in the acute phase being about six times higher on 4 dpi compared to 14 dpi and to healthy controls (Table 3). In addition, Hp reached extreme maxima at 4 dpi and 9 dpi, although this effect was not consistent in all infected calves. The kinetics of absolute BSA concentrations in BALF and blood of infected animals paralleled with concentrations being lower 4 and 9 dpi than 14 dpi (Fig. 2).

Multiple regression analysis revealed that only the concentrations of LBP and Hp in BALF supernatant were significantly linked to the respiratory score (Table 4, Fig. 3b). An additional significant effect of the BSA concentration in BALF was seen at 4 dpi, i.e. in the acute phase of infection only (Table 4).

**Table 4:** Significant relationships between absolute concentrations of APPs assessed in BALF supernatant and the respiratory score of calves with an experimentally induced respiratory *Chlamydia psittaci* infection

Dependent variable: respiratory score		
	All time points	4 dpi (acute phase)
Independent Variable	*P-value*	*P-value*
LBP in BALF	0.0000	0.2467
Hp in BALF	0.0026	0.0099
BSA in BALF	n.s.	0.0005
CRP in BALF	n.s.	n.s.
Lf in BALF	n.s.	n.s.

*LBP* lipopolysaccharide binding protein. *Hp* haptoglobin. *BSA* bovine serum albumin. *CRP* c-reactive protein. *Lf* lactoferrin. *P* probability level. *n.s*. not statistically significant (*P* > 0.05)

Legend to Table 4: Equations of multiple regressions

All time points:

$$ \begin{array}{l}\mathrm{Respiratory}\ \mathrm{Score} = 0.141 + 0.0217*\mathrm{L}\mathrm{B}\mathrm{P}\ \left[\mathrm{ng}/\mathrm{mL}\ \mathrm{B}\mathrm{ALF}\right] + 0.0003*\mathrm{H}\mathrm{p}\ \left[\mathrm{ng}/\mathrm{mL}\ \mathrm{B}\mathrm{ALF}\right]\\ {}\left(\mathrm{R}{}^2 = 55.26\%;P\hbox{-} Value\ \mathrm{of}\ \mathrm{the}\ \mathrm{model} = 0.0000;\ \mathrm{n}=39\right)\end{array} $$

Acute phase (4dpi):

$$ \begin{array}{l}\mathrm{Respiratory}\ \mathrm{S}\mathrm{core} = 0.772\ \hbox{--}\ 0.0067*\mathrm{L}\mathrm{B}\mathrm{P}\ \left[\mathrm{ng}/\mathrm{mL}\ \mathrm{B}\mathrm{A}\mathrm{LF}\right]\ \hbox{--}\ 0.0007*\mathrm{H}\mathrm{p}\ \left[\mathrm{ng}/\mathrm{mL}\ \mathrm{B}\mathrm{A}\mathrm{LF}\right] + \\ {}\ 0.1562*\mathrm{B}\mathrm{S}\mathrm{A}\ \left[\mathrm{ng}/\mathrm{mL}\ \mathrm{B}\mathrm{A}\mathrm{LF}\right]\\ {}\left(\mathrm{R}{}^2 = 56.24\%;P\hbox{-} Value\ \mathrm{of}\ \mathrm{the}\ \mathrm{model} = 0.0000;\ \mathrm{n}=13\right)\end{array} $$

**Fig. 5:**
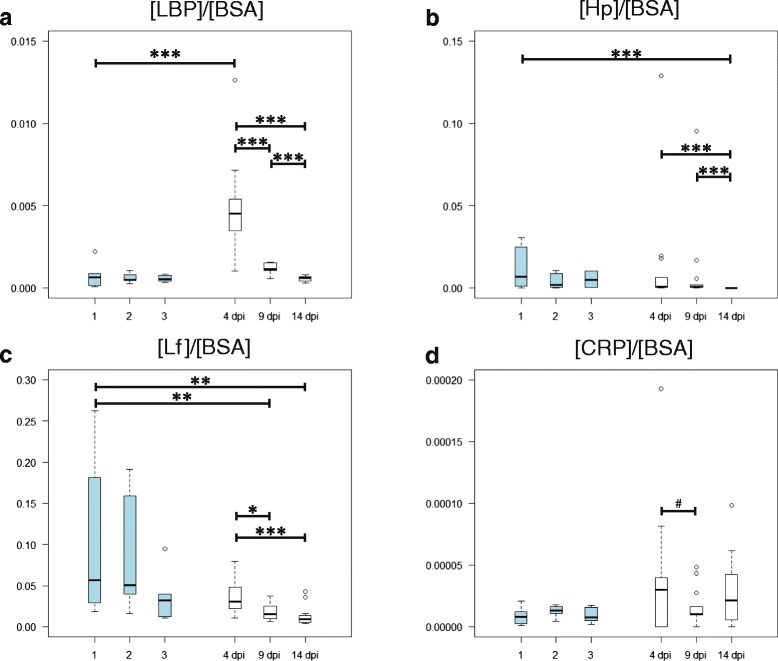
Quotients between [local marker]/[BSA] in BALF supernatant of healthy and *Chlamydia psittaci* infected calves. Values of infected animals were compared to the first sample from healthy controls (Mann–Whitney-*U* test with Holm adjustment). Lf: lactoferrin; BSA: bovine serum albumin; CRP: C-reactive protein; LBP: lipopolysaccharide binding protein; Hp: haptoglobin; dpi: days after inoculation; 1, 2, 3: first, second, and third sample from healthy controls; white: animals after inoculation with *C. psittaci* (*n* = 13); light blue: healthy control animals (*n* = 6); #: 0.05 ≤ *P* < 0.1; *: 0.01 ≤ *P* < 0.05; ** 0.001 ≤ *P* < 0.01; *** *P* < 0.0001

Considering the quotients between the given local marker and BSA in BALF [local marker]/[BSA], no significant differences were detected in between the different sampling points in healthy control animals (Fig. 5). In infected animals 4 dpi, the [LBP]/[BSA]-quotient was significantly increased as compared to 9 and 14 dpi and to healthy controls and it was significantly higher 9 than 14 dpi (Fig. 5a). The median of the [LBP]/[BSA]-quotient 4 dpi was six times higher than in healthy controls and more than eight times higher than in infected animals 14 dpi. The value of the [Hp]/[BSA]-quotient in infected animals 14 dpi was significantly lower than on 4 and 9 dpi and in healthy controls (Fig. 5b). The [Lf]/[BSA]-quotient in BALF supernatant of healthy controls and infected animals 4 dpi was significantly higher than in infected animals 9 and 14 dpi (Fig. 5c). Significant differences of the values of the [CRP]/[BSA]-quotients were detected neither between healthy controls and infected animals nor between different time points in infected animals (Fig. 5d). Nevertheless, there was a tendency for a decrease of the [CRP]/[BSA]-quotient from 4 to 9 dpi.

### Correlations of LBP and BSA between BALF supernatant and blood serum

For LBP, a significant rank correlation was found between the concentrations in BALF and the ones in blood (r_SP_ = 0.48; *P* = 0.0031) when all time points were included in statistical analysis. However, considering time points separately, no statistically secured correlations were seen any more between blood-LBP and BALF-LBP. Concentrations of BSA assessed in either blood or BALF were not statistically related at any time point.

## Discussion

In former studies, the bovine model of acute respiratory infection after inoculation with 10^8^ ifu *C. psittaci* was well characterized in terms of clinical and pathological outcome, various parameters of local and systemic inflammatory response, lung function, acid base status, and spreading and shedding of the pathogen [33, 30, 29, 28]. All animals developed signs of acute respiratory disease with fever within 36 h pi, with a maximum on 2 and 3 dpi. Clinical signs resolved within one week. Changes in the peripheral blood count were dominated by an increase of neutrophilic granulocytes with a regenerative left shift, followed by leukopenia. From 2 to 9 dpi, increased amounts of neutrophilic granulocytes were found in BALF. Histopathologically, fibrinopurulent bronchopneumonia with multifocal areas of necrosis and pleuritis was seen in the lungs of infected animals. With respect to systemic APPs, the pathophysiologic features mentioned above were accompanied by significant increases of LBP and Hp concentrations in the blood shortly after inoculation (compare Table 1).

In the present study, our goal was to analyze whether LBP, Hp, Lf, and CRP were secreted into broncho-alveolar fluid and thus, might be suitable as local biomarkers of respiratory inflammation in cattle utilizing the described bovine model of inrabronchial inoculation with *C. psittaci*. As expected, the respiratory score of all animals was significantly elevated during the first week after inoculation. In blood samples of these animals, we found significant changes in LBP-, albumin- and α-,β- and γ-globulin concentrations. In supernatants of BALF, we could verify changes in the absolute concentrations and in the [local marker]/[BSA]-quotients for LBP, Hp, and Lf, which paralleled the clinical course as well as signs of local and systemic inflammation. Absolute concentrations of LBP and Hp in BALF were even significantly associated with the severity of clinical signs.

In blood serum, albumin, fractions of globulins, and concentration of LBP showed the kinetics of a typical acute phase reaction [39–41]. The APP LBP showed an increase which paralleled the course of the respiratory score. Levels of the negative APP albumin dropped after onset of chlamydial infection in all animals. Blood levels of both, BSA and LBP correlated with clinical signs, with the correlation of BSA being stronger than the one of LBP. This interesting fact underlines the known importance of BSA and LBP as systemic APPs and markers of inflammation in bovines. The decreased albumin/globulin-quotient resulted from reduced albumin and increased α-globulin concentrations. Raised concentrations of α-globulins after infection were due to an increase of acute phase proteins such as α1-antitrypsin, Hp and α2-macroglobulin, which are major components of this fraction. The decrease of the concentration of the β-globulin fraction might result from a decrease of transferrin concentrations. Transferrin is the main part of the β-globulin fraction and considered a negative acute phase protein in cattle [42]. Thus the acute phase reaction in calves upon chlamydial infection could hereby be confirmed.

In BALF supernatant, the kinetics of albumin levels were very similar to blood. Since their actual concentrations showed high variability—probably due to the variability of the amount of fluid recovered during lavage and on the actual area of the lung that was lavaged [43, 44]—we tried to harmonize the measured values by normalization. Since no other origin than the liver is known, albumin could be a suitable marker for the degree of plasma leakage, *i.e.,* the inflammatory increase of permeability of lung endo—and epithelia. While the correlation between albumin concentration in blood and clinical signs was present throughout the whole study, albumin concentrations in BALF only correlated with clinical signs in the acute phase of the disease (4 dpi). This confirms the suitability of albumin concentrations in BALF for the normalization of the concentrations of local markers in BALF as performed in this study. When the ratio of [local marker]/[BSA] remains constant throughout the time interval analyzed, the local marker can be considered solely as blood derived, whereas local production of the marker leads to an increase of this ratio. For LBP and Lf, the time dependent course was more distinct for the [local marker]/[BSA] ratio than for the local concentration alone.

Of all analyzed local markers in the BALF supernatant of *Chlamydia psittaci* infected calves, the most significant changes were seen for LBP, which was markedly increased in animals 4 dpi. This correlates with our experience, that LBP in the blood of calves is a very reliable acute phase marker in respiratory disease [30, 27, 31, 45]. Increased LBP-levels have been reported in the BALF of pigs after viral infection [18, 46] and it was shown *in vitro* that pneumocytes of type II are capable of producing LBP [22], all supporting the hypothesis, that LBP can be produced in the lung as a local reaction to the inoculation with *C. psittaci*. In contrast to the other local markers analyzed in this study, LBP concentrations were significantly higher in infected animals than in healthy controls, and their variability is rather low. The most distinct changes were visible during the acute phase of the disease. Furthermore, concentrations of LBP in the BALF parallel the respiratory score and the systemic acute phase reaction as represented by LBP-, albumin- and globulin-concentrations in the blood. Together with its correlation with the respiratory score, this makes LBP the most promising candidate for a local marker of respiratory inflammation in cattle.

Hp is a major APP frequently investigated in cattle and showed increased serum concentrations upon respiratory bacterial and viral infection [45, 47, 48]. Hp concentrations were also shown to increase in the mammary gland [8] and in the broncho-alveolar fluid [19] upon local infection. Hp has already been detected in the BALF of calves inoculated with *Pasteurella haemolytica* [49]. Also, it could be shown that the haptoglobin gene is upregulated in the inflamed human lung [50]. In contrast to these results, in *C. psittaci* infected calves the [Hp]/[BSA]-quotient in BALF showed a time dependent decrease. Moreover, the [Hp]/[BSA]-quotient in healthy animals was as high as in diseased animals 4 dpi, not allowing discrimination of the latter group. However, there is a significant relationship between Hp concentration in BALF supernatant and the respiratory score of animals inoculated with *C. psittaci,* suggesting that there is an association between disease severity and amount of Hp in the lung. Further investigations with larger numbers of animals are needed to investigate this aspect and to clarify, whether Hp is a useful local marker for bovine lung disease.

Generally, Lf concentrations have been shown to correlate with the present numbers of neutrophils in body fluids [51–53]; therefore Lf concentrations in BALF were considered a possible local biomarker of inflammation. Lf is the only protein analyzed in this study with concentrations much higher in BALF supernatant of healthy and diseased animals than in the blood of healthy calves (cf. Tables 1 and 2), strongly supporting the local production of this protein. Resident neutrophilic granulocytes are the most likely source of Lf in lung [54]. The process of broncho-alveolar lavage leads to very mild focal alteration of the lung [55], being a possible instant stimulus for degranulation and increasing the local Lf concentration. The [Lf]/[BSA]-quotients measured here were similar in healthy controls and infected animals 4 dpi and dropped towards the end of our study. Since the increases of Lf-levels in blood are maximal already 12 h after inoculation with *Mannheimia haemolytica* in calves (Schroedl, unpublished data) it is possible that after inoculation with *C. psittaci* even higher levels of Lf might be detectable in BALF much earlier than 4 dpi. This could mean that local Lf is depleted in the course of the infection, and that initial Lf levels in BALF represent a local defense mechanism due to its antibacterial and antiviral properties [56]. Due to the increased anesthetic risk in animals with systemic disease and dyspnea, no bronchoscopy was performed earlier than 4 dpi in the present study. To clarify whether Lf concentrations in supernatant of BALF would increase at earlier time points, further studies with BALF sampling early after inoculation would be necessary.

CRP concentrations in blood were poorly correlated with inflammatory disease in cattle [57, 58, 30]. Nevertheless, CRP is involved in the local inflammatory response of the udder in cows and was suggested as a valuable parameter of mastitis [59]. In the lung defense mechanisms, CRP does not seem to play any role in bovines, since the [CRP]/[BSA]-quotient was not significantly affected by inoculation with *C. psittaci* in the present study.

In some cases the variation of local marker concentrations measured in BALF was rather high. However, extreme values could not be associated with a different clinical score in these animals since the course of the infection in this large animal model is very homogenous with all individuals showing a comparable severity of respiratory disease with fever [33, 30, 29].

BALF samples obtained 14 dpi were gained *post mortem* from the exenterated lung. This methodological difference might have influenced the concentrations of local markers analyzed in BALF supernatant. It was not the aim of the present study to evaluate methodical influences on the parameters presented; this must be subject of further studies. Yet this aspect should be considered, since depending on the animal species and the experimental setting, the method of sampling broncho-alveolar lavage differs.

The association of increased APP production in the lung with the presence of inflammation has been shown in previous studies in humans, mice and pigs [60–62, 50, 46, 18, 19]. A study in calves could show higher concentrations of Hp and SAA in the BALF of animals with non-experimental bronchopneumonia as compared to healthy controls, selection of calves was based on clinical signs [26]. In our study, we investigated this aspect in a well-defined large animal model of respiratory infection with *C. psittaci* with known outcome. We could confirm that changes in APP concentrations in the BALF were associated with local inflammation and we could show that the concentration of LBP in BALF strongly paralleled the kinetics of the respiratory score and the systemic acute phase response. In contrast to the results of Coskun et al. [26], we could not confirm the local increase of Hp concentrations in the lung. However, we did see a significant relationship between local Hp concentration and clinical signs, underlining again the importance of further investigations of the local role of Hp in bovine respiratory disease.

## Conclusion

The acute phase reaction in the bovine model of respiratory *C. psittaci* infection could be confirmed by the kinetics of total protein, albumin, and concentrations of various fractions of globulins in the blood serum. LBP seems to be a promising local biomarker in the lung of calves, since its kinetics were time dependent on the course of the infection and LBP concentration as well as [LBP]/[BSA]-quotient were much higher in infected animals than in healthy controls. Also, there was a significant relationship between BALF concentration of LBP and clinical signs. Hp and Lf showed a time-dependent decrease in the BALF of infected animals, but absolute concentrations as well as [local marker]/[BSA]-quotients were comparably high in healthy controls and in infected animals 4 dpi, and only concentrations of Hp could be correlated to clinical signs. CRP was not considered a valuable local biomarker in bovine respiratory infections since the concentrations and [CRP]/[BSA]-quotients were similar in healthy and infected animals and did not show any time-dependent course.

## Abbreviations

APP(s): Acute phase protein(s)
BALF: Broncho-alveolar lavage fluid
BSA: Bovine serum albumin
*C.*: *Chlamydia*
CRP: C-reactive protein
dpi: days post inoculation
Hp: Haptoglobin
LBP: Lipopolysaccharide binding protein
Lf: Lactoferrin
OD: Optical density
PBS: Phosphate-buffered saline
SAA: Serum amyloid A
